# Platelet‐rich fibrin‐based matrices to improve angiogenesis in an *in vitro* co‐culture model for bone tissue engineering

**DOI:** 10.1002/term.2475

**Published:** 2017-08-30

**Authors:** Eva Dohle, Karima El Bagdadi, Robert Sader, Joseph Choukroun, C. James Kirkpatrick, Shahram Ghanaati

**Affiliations:** ^1^ FORM, Frankfurt Oral Regenerative Medicine, Clinic for Maxillofacial and Plastic Surgery Johann Wolfgang Goethe University Frankfurt am Main Germany; ^2^ Pain Therapy Center Nice France; ^3^ Department of Biomaterials Gothenburg Sweden

**Keywords:** angiogenesis, bone tissue engineering, endothelial progenitor cells, inflammation, platelet rich fibrin, wound healing

## Abstract

In the context of prevascularization strategies for tissue‐engineering purposes, co‐culture systems consisting of outgrowth endothelial cells (OECs) and primary osteoblasts (pOBs) have been established as a promising *in vitro* tool to study regeneration mechanisms and to identify factors that might positively influence repair processes such as wound healing or angiogenesis. The development of autologous injectable platelet‐rich fibrin (PRF), which can be generated from peripheral blood in a minimal invasive procedure, fulfils several requirements for clinically applicable cell‐based tissue‐engineering strategies. During this study, the established co‐culture system of OECs and pOBs was mixed with injectable PRF and was cultivated *in vitro* for 24 h or 7 days. The aim of this study was to analyse whether PRF might have a positive effect on wound healing processes and angiogenic activation of OECs in the co‐culture with regard to proinflammatory factors, adhesion molecules and proangiogenic growth factor expression. Histological cell detection revealed the formation of lumina and microvessel‐like structures in the PRF/co‐culture complexes after 7 days of complex cultivation. Interestingly, the angiogenic activation of OECs was accompanied by an upregulation of wound healing‐associated factors, as well as by a higher expression of the proangiogenic factor vascular endothelial growth factor, which was evaluated both on the mRNA level as well as on the protein level. Thus, PRF might positively influence wound healing processes, in particular angiogenesis, in the *in vitro* co‐culture, making autologous PRF‐based matrices a beneficial therapeutic tool for tissue‐engineering purposes by simply profiting from the PRF, which contains blood plasma, platelets and leukocytes.

## 1 INTRODUCTION

A principal challenge in the field of regenerative medicine is the achievement of the best possible tissue regeneration and effective wound healing after surgery in large soft tissue and bone defect areas. Therefore, the development of tissue‐engineered constructs undergoes permanent optimization, with different strategies being developed to fulfil the clinical need of successful tissue regeneration. One essential and major requisite for successful bone and soft tissue regeneration is sufficient vascularization in order to supply the region of interest, i.e. the defect, with nutrients and oxygen. In order to improve the process of neovascularization in damaged or engineered tissues, different vascularization strategies have been established and include functionalization of the scaffold (Lovett, Lee, Edwards, & Kaplan, 2009; Santos et al., 2008) and the development of drug delivery systems for proangiogenic growth factors (Geiger et al., 2005; Gu, Amsden, & Neufeld, 2004). In addition, prevascularization techniques using stem cell‐based therapies (Rivron, Liu, Rouwkema, de Boer, & van Blitterswijk, 2008; Rouwkema, de Boer, & Van Blitterswijk, 2006) or the combination of a construct with endothelial cells/endothelial progenitor cells and complex co‐culture systems to generate a prevascularized tissue are also under investigation (Grellier et al., 2009; Rouwkema et al., 2006; Tabata, Miyao, Yamamoto, & Ikada, 1999) in order to enhance the vascularization process of tissue‐engineered constructs. For these complex co‐culture strategies, different endothelial cell types, such as human umbilical vein endothelial cells or human dermal microvascular endothelial cells have been used in combination with osteoblasts or mesenchymal stem cells mimicking the microenvironment that can be found *in vivo* (Stahl et al., 2004; Unger, Dohle, & Kirkpatrick, 2015; Unger, Halstenberg, Sartoris, & Kirkpatrick, 2011; Unger et al., 2010; Villars et al., 2002). Nevertheless, the sources of mature endothelial cells, e.g. the umbilical cord or the juvenile foreskin, are making the translation of those co‐cultures from bench to bedside practically impossible.

Therefore, a very promising autologous cell source for proangiogenic therapies are endothelial progenitor cells, in particular late outgrowth endothelial cells (OECs), which meet a number of criteria that are essential for a possible clinical application (Hristov, Zernecke, Liehn, & Weber, 2007; Kim & von Recum, 2008; Rafii & Lyden, 2003). They can be easily isolated from the peripheral blood in a minimally invasive procedure, and reveal a high proliferation potential as well as a marked angiogenic capability in proangiogeneic matrices *in vitro* (Amini, Laurencin, & Nukavarapu, 2012; Gulati et al., 2003; Yoon et al., 2005). In the context of endothelial progenitor cells to improve the vascularization process, co‐culture systems consisting of OECs and primary osteoblasts (pOBs) constitute a promising strategy and resulted in angiogenic activation of OECs and the formation of microvessel‐like structures *in vitro* as well as *in vivo* (Fuchs, Hofmann, & Kirkpatrick, 2007; Fuchs, Ghanaati, et al., 2009; Fuchs, Jiang, et al., 2009). However, these co‐cultures of OECs and pOBs still possess some limitations due to the fact that an additional proangiogenic stimulus is essential to achieve a timely induction of the neovascularization process *in vitro*. Previous studies have documented that rapid initiation of angiogenesis could only be achieved when co‐cultures were treated with proangiogenic growth factors like vascular endothelial growth factor (VEGF), morphogens or even with activated macrophages mimicking the natural responses of the human body (Dohle et al., 2010; Dohle et al., 2011; Dohle et al., 2014). Furthermore, OEC/pOB co‐cultures strongly require the co‐implantation of proangiogenic matrices like *Matrigel®* for cell growth and induction of fast microvessel‐like structure formation *in vivo* (Fuchs, Ghanaati, et al., 2009).

From the clinical point of view, an optimal prevascularized cell‐based tissue‐engineered construct should be of natural origin, completely autologous, biocompatible and it should promote the patient's own natural tissue regeneration. In addition, a clinically simple and fast preparation and application of a material would be very beneficial. Therefore, Choukron and colleagues started to develop a new innovative concept to overcome all previous clinical limitations in the field of tissue engineering and regenerative medicine (Choukroun, Schoeffler, & Vervelle, 2001; Choukroun et al., 2006a; Choukroun et al., 2006b; Dohan et al., 2006a; Dohan et al., 2006b; Ghanaati et al., 2014). The use of platelet‐rich fibrin (PRF) seems to comply with all the requirements of an excellent material for tissue‐engineering purposes by promoting a material‐induced tissue reaction that could lead to natural regeneration processes. In particular, the low speed centrifugation concept (LSCC) resulting in the generation of injectable PRF with enriched platelet and leukocyte concentration might be able to boost the process of wound healing in a highly effective way (Choukroun & Ghanaati, 2017). Leukocytes as well as platelets play a key role during the process of wound healing and tissue regeneration (Davis et al., 2014). Under physiological conditions, a fibrin clot consisting of aggregated platelets is formed, which closes the wound in a primary manner and initiates the inflammation phase through growth factor release and attraction of leukocytes to sites of injury (Tsaryk, Peters, Unger, Scharnweber, & Kirkpatrick, 2007; Werner & Grose, 2003). Platelets as well as activated leukocytes secrete different growth factors and proinflammatory cytokines as well as mediating endothelial cell adhesion, migration, proliferation and the formation of granulation tissue, which finally results in neovascularization and new tissue formation (Adams & Alitalo, 2007; Diegelmann & Evans, 2004).

In the present study, we investigated the *in vitro* effect of injectable PRF on regeneration processes in the established co‐culture system consisting of OECs and pOBs. The aim was to examine whether the simple addition of PRF would result in an induction of wound healing processes and might positively influence the process of angiogenesis via inflammatory processes in the co‐culture. Therefore, co‐cultures were mixed with injectable PRF for 24 h and 7 days before the cells were analysed in terms of inflammatory activation and angiogenesis formation using immunohistochemistry, enzyme‐linked immunosorbent assay (ELISA) and quantitative real‐time reverse transcription‐polymerase chain reaction (RT‐PCR).

## 2 MATERIALS AND METHODS

All cells that were used for this study were obtained from excess tissue and their application was in accordance with the principle of informed consent and approved by the responsible ethics commission of the state of Hessen, Germany.

### 2.1 Primary cells

Human OECs were isolated from peripheral blood buffy coats as previously described (Fuchs, Hermanns, & Kirkpatrick, 2006). The cells were grown until confluence on fibronectin‐coated (10 μg/ml, Millipore) 24‐well plates in EBM‐2 medium (Lonza, Basel, Switzerland), supplemented with EGM‐2 BulletKit, 1% penicillin/streptomycin (Sigma‐Aldrich, St. Louis, MO, USA) and additional 4% fetal bovine serum (Biochrom, Berlin, Germany). The cells were cultivated at 37°C in an atmosphere of 5% CO_2_ and 95% air. After 3–4 weeks, late OECs with a cobblestone‐like morphology, a typical mature endothelial marker profile and a high proliferation potential appeared. These cells were trypsinized and expanded on fibronectin‐coated 24‐well plates (10 μg/ml) over several passages using a splitting ratio of 1:2. For this study, OECs were used from passage 8 to passage 14. Human pOBs were isolated from cancellous bone fragments, as previously described (Hofmann et al., 2003) from healthy donors, and cultivated in Dulbecco's Modified Eagle's Medium Nutrient Mixture F‐12 (Sigma‐Aldrich), supplemented with 10% fetal bovine serum (Biochrom) + 1% penicillin/streptomycin (Sigma‐Aldrich) at 37°C in an atmosphere of 95% air and 5% CO_2_. Cells were passaged in a ratio of 1:2. pOBs were used in passage 3 for this study.

### 2.2 Cell culture experiments

**Figure 1:**
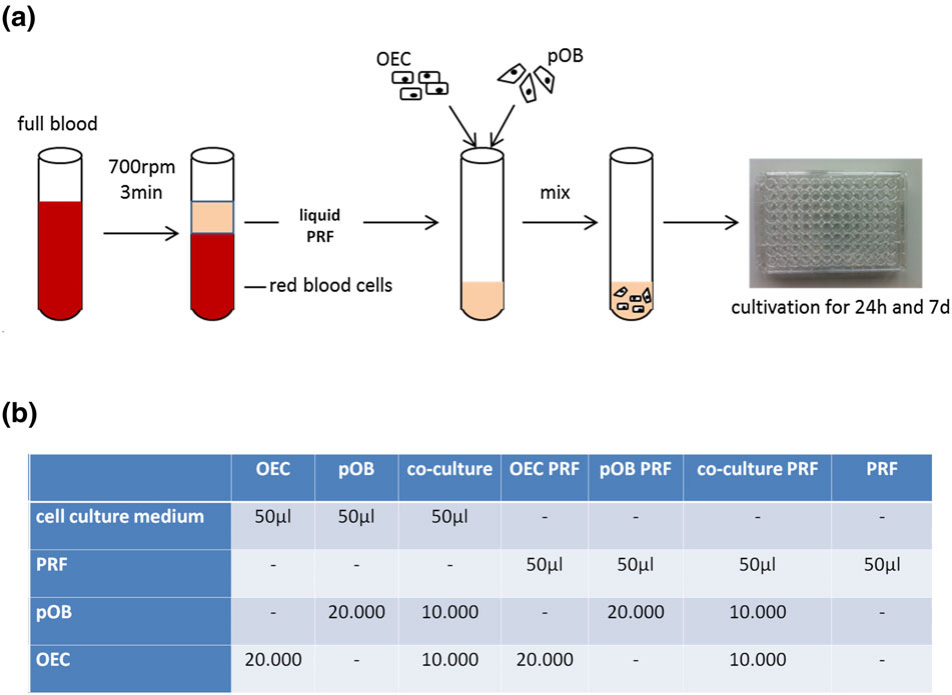
Schematic overview representing the experimental design and the analysed experimental groups. (a) Platelet‐rich fibrin was prepared using a low speed centrifugation concept and was mixed with the different primary cells before being transferred to 96‐well plates for further cultivation. (b) Overview of all tested experimental groups during the study [Colour figure can be viewed at http://wileyonlinelibrary.com]

Peripheral blood was collected from four healthy volunteers (three female and one male) and centrifuged in a Duo centrifuge (Process for PRF, Nice, France) with a fixed angle rotor with a radius of 110 mm for 3 min at 700 rpm according to a previously published study using LSCC (Choukroun & Ghanaati, 2017). All volunteer donors agreed to the informed consent, were free of infectious diseases and were not consuming alcohol or nicotine. For the purpose of these experiments, sterile uncoated plastic tubes (i‐PRF tubes, Process for PRF) with a volume of 10 ml were used to generate the blood concentrates via centrifugation (700 rpm, 3 min) as schematically depicted in Figure 1a. After centrifugation, blood had been divided into its components. Due to the fact that no chemical anticoagulant agents were used, fluid matrices were generated that allowed the mixing of the primary cells with the liquid PRF. The liquid PRF was assembled and quickly transferred to a 50 ml plastic tube (Figure 1a). Twenty thousand cells per well of monocultures (OEC monoculture, pOB monoculture) and 10 000 OECs +10 000 pOBs for co‐culture experiments were gently mixed with the liquid PRF and transferred to a 96‐well plate (Figure 1a, b). These experimental steps need to be carried out quickly due to time‐related clotting of the PRF/cell mixture. Subsequently, the PRF/cell mixtures were incubated for 1 h at 37°C to allow the clotting of the PRF/cell mixture before 150 μl cell culture medium (EBM‐2 plus supplements; Lonza) was added for an incubation time of 24 h and 7 days. As controls, co‐ and monocultures without PRF as well as pure PRF were also seeded on 96‐well plates (Figure 1b). After 24 h and 7 days of cultivation, the clots were fixed for immunohistochemistry. In addition, supernatants were collected for ELISA and RNA was isolated for quantitative real‐time PCR. During the course of cultivation, the cell culture medium was not changed. In total, three different donors were analysed during this study.

### 2.3 Immunohistochemistry

The samples were fixed in 4% buffered formalin (Roti*‐*Histofix 4% acid free pH 7, Carl‐Roth), removed from the 96‐well plates, dehydrated in an ascending ethanol series using a tissue processor (Leica TP1020, Wetzlar, Germany) and embedded in paraffin blocks. The samples were cut into histological sections with a thickness of 4 μm using a rotation microtome (Leica RM2255). For immunohistochemical staining the sections were rehydrated in a descending ethanol series, pretreated with citrate buffer at pH 6 for 20 min at 96°C and finally rinsed in distilled water before they were stained with mouse anti‐human CD31 (1:40, Dako, MO 08223) by Autostainer (Lab vision Autostainer 360, Thermo Fisher Scientific). Mouse‐specific secondary antibody was used (HRP Ultravision kit, Thermofisher) and visualization was detected using DAB (Dako). One slide of each experimental PRF group was stained with haematoxylin and eosin (H&E) for general evaluation of cell distribution and fibrin morphology. Histological examination was carried out using a light microscope (Nikon Eclipse 80i, Tokyo, Japan) and images were taken with a connected Nikon DS‐Fi1/digital camera and a Nikon digital sight unit DS‐L2. For immunofluorescent staining, rehydrated and sonicated sections were permeabilized with 0.5% Triton X/phosphate‐buffered saline (PBS) and washed three times with PBS before they were incubated with mouse anti‐human CD31 (1:40, Dako, MO 08223) or with goat anti‐human CD31 (1:50, Santa Cruz Biotechnology) and mouse anti‐human CD68 (1:200, Dako M0814) or with mouse anti‐human osteopontin (RTU) or with mouse anti‐human CD45 (1:100) diluted in a 1% bovine serum albumin/PBS solution for 60 min at room temperature. After washing three times with PBS, the cells were incubated with the secondary anti‐mouse antibody Alexa 488 (Molecular Probes, MoBiTec, Göttingen, Germany) diluted 1:1000 in a 1% bovine serum albumin/PBS solution for 60 min at room temperature, protected from light. The cells were mounted with Fluoroshield (ImmunoBioScience Corp., Mukilteo, WA, USA) and examined using a confocal laser scanning microscope (LeicaTCS‐NT, Leica Microsystems, Wetzlar, Germany).

### 2.4 Image quantification

The histochemically stained images (CD31) were analysed using the image processing software Fiji. Lumina structures lined with CD31‐positive staining were defined, selected and extracted from the remaining images. The lumen area (%) was calculated from the total area of the images. For image quantification, three images of each experimental group were analysed. All calculations were performed using MS Excel (Microsoft Office, Microsoft, Munich, Germany).

### 2.5 ELISA

Culture supernatants of the different experimental groups were collected after 24 h and after 7 days of cultivation. The concentrations of VEGF, platelet derived growth factor (PDGF‐BB), E‐selectin and intercellular adhesion molecule‐1 (ICAM‐1) were measured using DuoSet® ELISA Development Systems according to the manufacturer's protocol (R&D Systems). A streptavidin‐horseradish peroxidase colorimetric reaction was used to visualize protein concentrations and the optical density of each well was measured using a microplate reader (Tecan, Crailsheim) at a wavelength of 450 nm. The results are demonstrated as absolute values as indicated in the relevant figure.

### 2.6 Quantitative real‐time RT‐PCR

RNA was isolated using TRIZOL reagent (Sigma). Thus, three wells of each experimental group of a 96‐well plate was assembled in 500 μl TRIZOL in one 1.5 ml tube and incubated for 5 min at room temperature. Chloroform (200 μl) was added to each tube, followed by vortexing for at least 15 s. After an incubation time of 3 min, tubes were centrifuged for 15 min at 12 000×*g* at 4°C. The aqueous phase containing the RNA was removed and transferred to a new tube before 500 μl isopropanol was added for RNA precipitation. After another centrifugation step (12 000×*g*, 4°C, 10 min), supernatant was removed, the RNA pellet was washed in 1 ml ethanol and centrifuged again (7500×*g*, 4°C, 5 min). The pellet was dried and resolved in 10 μl RNAfree water before the RNA concentration was measured using a nanodrop spectrophotometer (NanoDrop, Wilmington). Extracted RNA (1 μg) was used for reverse transcription by using an Omniscript RT kit according to the manufacturer's protocol (Quiagen). For quantitative real‐time PCR the following primers were used for this study: VEGF‐A, ICAM‐1, PDGF‐BB, E‐selectin, bone morphogenic protein 2 (BMP‐2) and alkaline phosphatase (ALP) (all obtained from Quiagen). 60S ribosomal protein L13A (RPL13A) was used as endogenous control. cDNA (4 ng) was used for one reaction. Quantitative real‐time PCR was performed in triplicate with the following cycler programme: 95°C for 10 min, 95°C for 15 s and 60°C for 1 min, 40 cycles. To specify the length of the DNA fragments a dissociation stage was added to the programme: 95°C for 15 s, 60°C for 1 min, 95°C for 15 s and 60°C for 15 s. Relative gene expression was determined using the ∆∆Ct method. Gene expression was compared by setting control cultures to 1 (reference value) as indicated in the relevant figures.

### 2.7 Statistical analyses

All experiments were performed with at least three different donors. The data are presented as mean values ± standard deviation. Statistical significance was evaluated using the paired Student's *t*‐test. Statistical analyses were performed with MS Excel and significance was assessed by *p*‐value <0.03 or *p*‐value <0.05, respectively.

## 3 RESULTS

### 3.1 Angiogenic activation of OECs in co‐culture‐PRF complex

**Figure 2:**
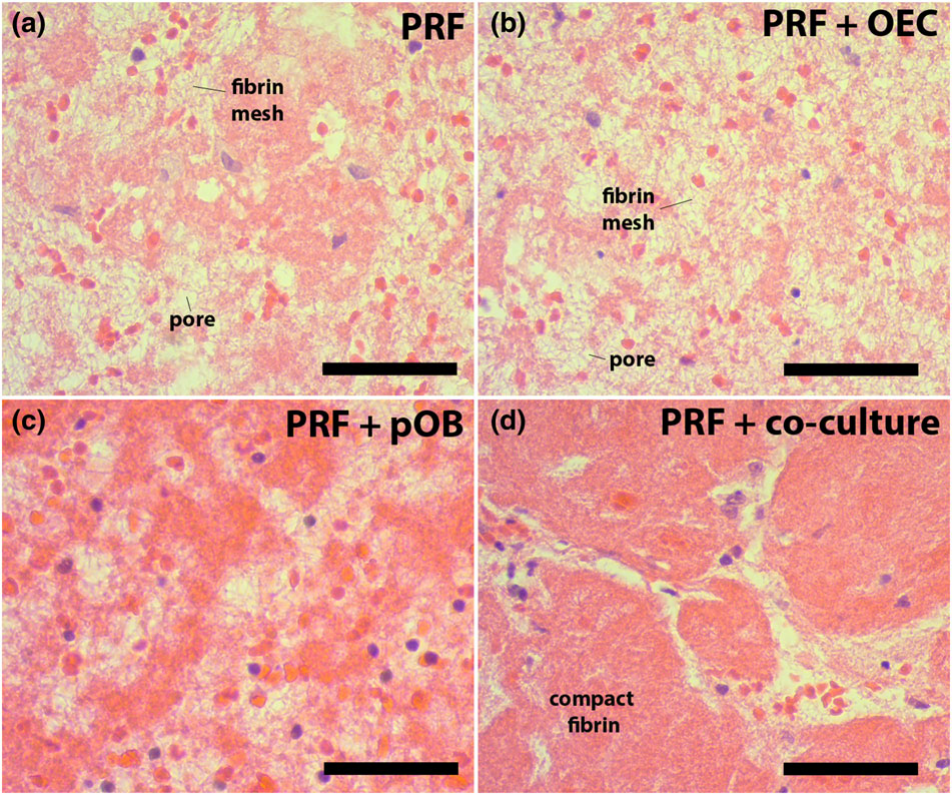
Cell distribution and fibrin morphology in different platelet‐rich fibrin (PRF) clots. Injectable PRF was cultivated for 24 h (data not shown) and for 7 days, before the clots were fixed, embedded in paraffin, cut into 4 μm sections and stained with haematoxylin and eosin stain (a). Cell distribution and fibrin morphology in PRF clots alone was compared with PRF mixed with outgrowth endothelial cells (OECs) (b), primary osteoblasts (pOBs) (c) or OECs and pOBs (d). Scale bars: 75 μm (a–d). *n* = 3 [Colour figure can be viewed at http://wileyonlinelibrary.com]

**Figure 3:**
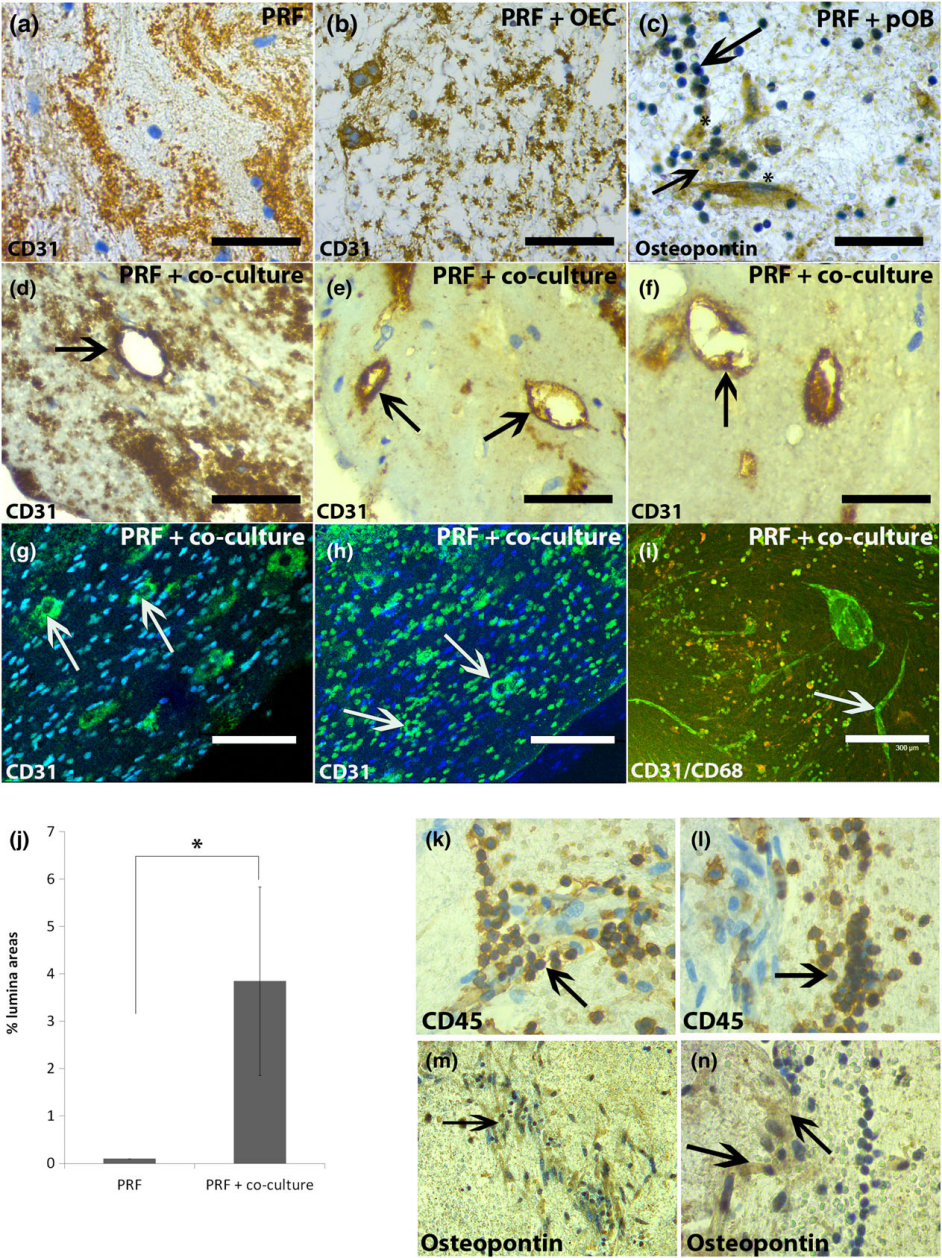
Lumina and microvessel‐like structure formation in co‐cultures mixed with platelet‐rich fibrin (PRF). Injectable PRF was mixed with either outgrowth endothelial cells (OECs) (b), primary osteoblasts (pOBs) (c) or co‐cultures of both cell types and clot/cell mixture was cultivated for 24 h (data not shown) and 7 days before they were embedded in paraffin, cut into 4 μm sections and stained immunohistochemically (a–f) and immunofluorescently (g, h) for the endothelial marker CD31 and for the osteoblastic marker osteopontin (c). Additionally, whole PRF/co‐culture clots were stained immunofluorescently for CD31 and CD68 after 7 days of cultivation (i). The histological sections of the PRF/cell complexes were additionally stained alternately for CD45 (k, l) and osteopontin (m, n) revealing the existence of CD45‐positive cells next to osteopontin‐positive cells (k–m). Scale bars: 75 μm (a–f); 150 μm (g, h); 300 μm (i). *n* = 3 [Colour figure can be viewed at http://wileyonlinelibrary.com]

In general, injectable PRF represents a stable three‐dimensional fibrin‐based matrix for primary cells that were seeded inside the clots and cultivated for 24 h and 7 days. Histological staining for H&E, visually evaluating the cell distribution and the fibrin structure after 7 days of cultivation, showed that the fibrin network of PRF clearly differed in their porous structure between the different experimental groups (Figure 2a–d). The fibrin structure seemed to be markedly compact when pOBs in monoculture or co‐cultures of OECs and pOBs were mixed with the clot (Figure 2c, d) compared with pure PRF or PRF/OEC complexes, where the fibrin structure seemed to be more porous (Figure 2a, b). However, OEC as well as pOB monocultures could be found in all parts of the clot and were distributed in a balanced manner after both time points of cultivation, whereas endothelial cells in monoculture were commonly organized in aggregations of one or two cells after 7 days of cultivation, respectively, as demonstrated by a positive staining for the endothelial cell marker CD31 (Figure 3b, arrow). When pOB in monoculture were mixed with PRF and cultivated for 7 days, monocytic‐like cells clearly accumulated at sites of osteopontin‐positive cells, indicating the pOBs in the clot (Figure 3c). Furthermore, the histological sections of the PRF/cell complexes were additionally stained alternately for CD45 (Figure 3l) and osteopontin (Figure 3n) revealing the existence of CD45‐positive cells next to osteopontin‐positive cells (Figure 3k–m). Immunohistochemical staining for the endothelial cell‐specific marker CD31 could not reveal any evidence for an ongoing angiogenic process in pure PRF clots compared with PRF clots combined with pOB or OEC monocultures after 24 h (data not shown) and after 7 days of cultivation (Figure 3a–c). In contrast, when both cell types were co‐cultivated in PRF clots, the OECs seemed to be angiogenically activated, as indicated by a strongly positive staining for CD31 of OECs, which clearly formed vessel‐like lumina after 7 days of co‐cultivation in the fibrin‐based scaffold (Figure 3d–h, arrows). Vessel‐like lumina CD31‐positive stained structures were quantified in the appropriate clots and areas of lumina structures were determined in co‐cultures cultivated in the PRF clot and compared with co‐cultures alone (Figure 3j). Significantly more lumina areas could be found in co‐cultures cultivated in the fibrin clot. To additionally visualize the PRF‐mediated formation of microvessel‐like structures in the co‐cultures consisting of OECs and pOBs, whole co‐culture/PRF clots were also stained immunofluorescently for the endothelial cell‐specific marker CD31 as well as for the monocytic marker CD68 (Figure 3i). After 7 days of PRF/co‐culture cultivation, a considerable number of angiogenic structures could be detected, thus confirming the proangiogenic effect of PRF on the OECs in the co‐culture system (Figure 3i, arrow). CD31‐positive cells, representing the OECs in this complex, clearly arranged themselves to form microvessel‐like structures close to the PRF surface area, which could be visualized using a confocal laser scanning microscope (Figure 3i).

### 3.2 Injectable PRF activates wound healing processes in the co‐culture system consisting of pOBs and OECs

**Figure 4:**
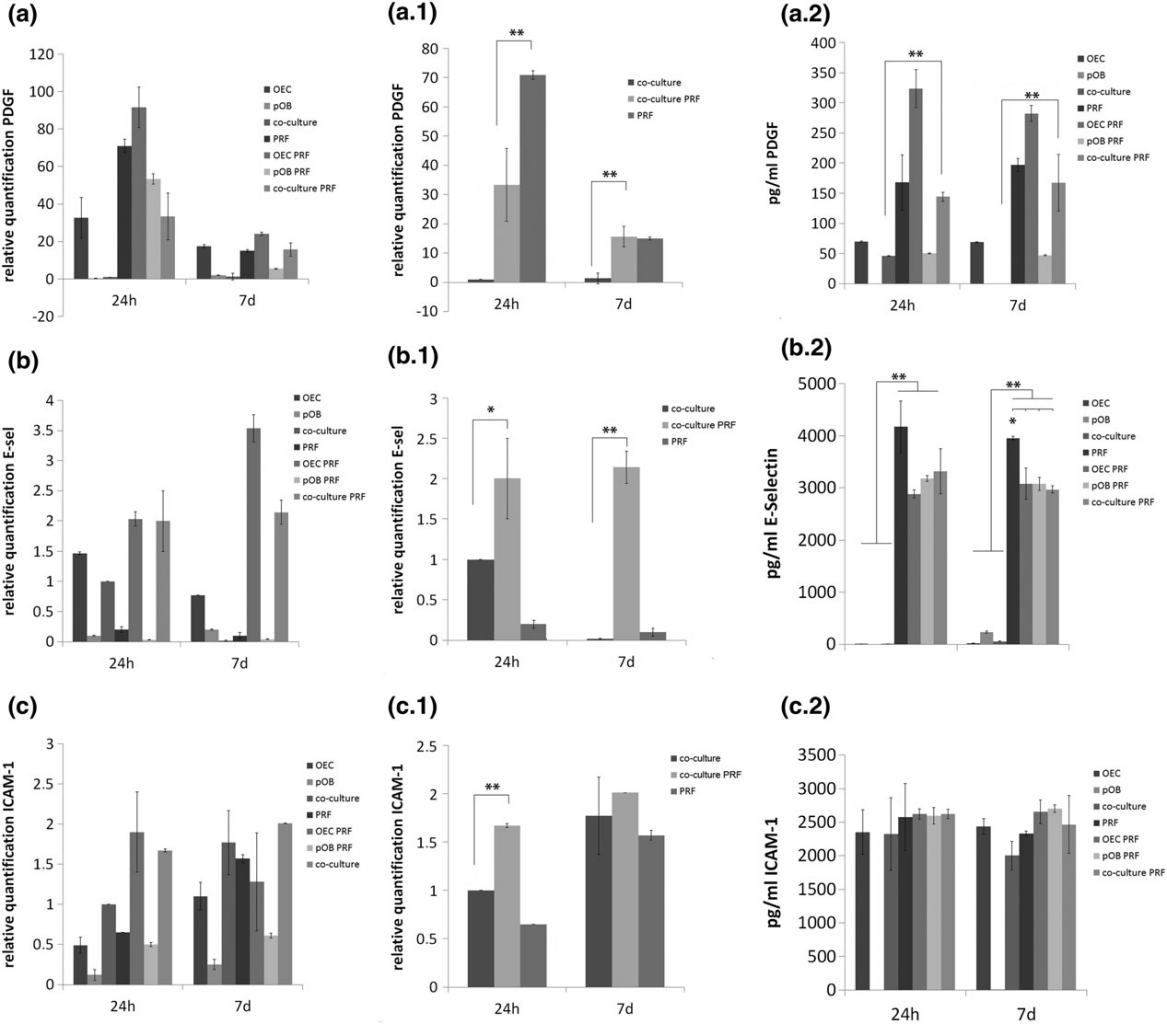
Gene expression analysis and determination of protein production of wound healing‐associated molecules platelet derived growth factor (PDGF), intercellular adhesion molecule‐1 (ICAM‐1) and E‐selectin in different platelet‐rich fibrin (PRF) clots. Injectable PRF was mixed with either outgrowth endothelial cells (OECs), primary osteoblasts (pOBs) or co‐cultures of both cell types and clot/cell mixture was cultivated for 24 h and 7 days before relative gene expression of PDGF (a, a.1), E‐selectin (b, b.1) and ICAM‐1 (c, c.1) were determined using quantitative real‐time polymerase chain reaction and supernatants were analysed for protein secretion (a.2, b.2, c.2). (a–c) General overview comparing gene expression of wound healing‐associated factors in PRF, monocultures, co‐cultures and PRF/cell clots. (a.1, b.1, c.1) Comparative gene expression analysis of PDGF, E‐selectin and PDGF in co‐cultures with and without the addition of PRF. Statistical significance was assessed when *p < 0.05 and **p < 0.03. *n* = 3

In order to analyse whether the cultivation of PRF with the co‐culture of pOBs and OECs might catalyse the initiation of wound healing mechanisms that might finally lead to the angiogenic activation of OECs in the co‐culture/PRF clot, RNA was extracted from the clots and the gene expression levels of factors that are involved in wound healing, including PDGF, E‐selectin and ICAM‐1, were compared after 24 h and after 7 days of cultivation using quantitative real‐time PCR (Figure 4a.1, b.1, c.1). Due to the fact that this study also intended to obtain a conception about the potential origin of the analysed factors, relative gene expression levels of all appropriate control cultures were additionally analysed (Figure 4a–c). PDGF, an important component in the initiation of wound repair, is generally expressed in OEC monocultures, in pure PRF as well as in the combination of these experimental groups after 24 h and after 7 days of cultivation, whereas a higher expression of PDGF could be detected after 24 h of cultivation in these groups (Figure 4a). Comparing the relative gene expression of PDGF in pure co‐cultures with co‐cultures cultivated in the PRF clot, a significantly higher expression was clearly demonstrated in the co‐culture/PRF clots after both cultivation time points (Figure 4a.1). After 7 days of co‐culture/PRF cultivation, the relative gene expression of PDGF was upregulated more than 30 times compared with the expression of this factor in the co‐culture alone. These results evaluated on the gene expression level are in accordance with data from PDGF protein secretion determination evaluated using ELISA (Figure 4a.2). PDGF protein secretion could not be found in pOB monocultures but in all other experimental group with a general trend of producing more PDGF when cells were mixed with PRF, with a significant difference when comparing PDGF production in co‐cultures with PDGF secretion in co‐culture/PRF complexes (Figure 4a.2). Similar results could be obtained for the relative gene expression of E‐selectin and ICAM‐1, essential adhesion molecules that are important for the interaction of endothelial cells with leukocytes during wound healing. E‐selectin is generally expressed by endothelial cells and by endothelial cells combined with PRF, but less expressed by pOBs (Figure 4b). The difference in relative gene expression of E‐selectin in co‐cultures compared with co‐cultures combined with PRF could be assessed as statistically significant and revealed a significantly higher gene expression of E‐selectin when the co‐culture was mixed with PRF (Figure 4b.1). After 7 days of PRF/co‐culture cultivation the expression of E‐selectin was significantly upregulated more than two times (Figure 4b.1). Additional protein secretion analysis revealed that E‐selectin is less secreted by OEC‐ and pOB monocultures or co‐cultures of both cell types but is highly secreted by PRF alone and can also be found in all experimental groups when combined with PRF. This could be assessed as statistically significant (Figure 4b.2). In addition, the expression of ICAM‐1, usually responsible for transmigration of leukocytes during wound repair, was also clearly upregulated in co‐culture/PRF complexes compared with the expression of this adhesion molecule in the co‐culture of OECs and pOBs alone, whereas statistical significance could only be determined for the 24 h time point of cultivation (Figure 4c.1). In general, ICAM‐1 expression was expressed in OEC, their co‐culture, in pure PRF as well as in their combination, but less in pOB monocultures (Figure 4c.1). ICAM‐1 protein secretion could be determined in all experimental groups but was not secreted by pOBs in monoculture, as evaluated via ELISA and depicted in Figure 4c.2. Nevertheless, PRF seems to have no effect on ICAM‐1 protein secretion in general.

### 3.3 Injectable PRF‐mediated effect on VEGF expression in co‐cultures of OECs and pOBs

**Figure 5:**
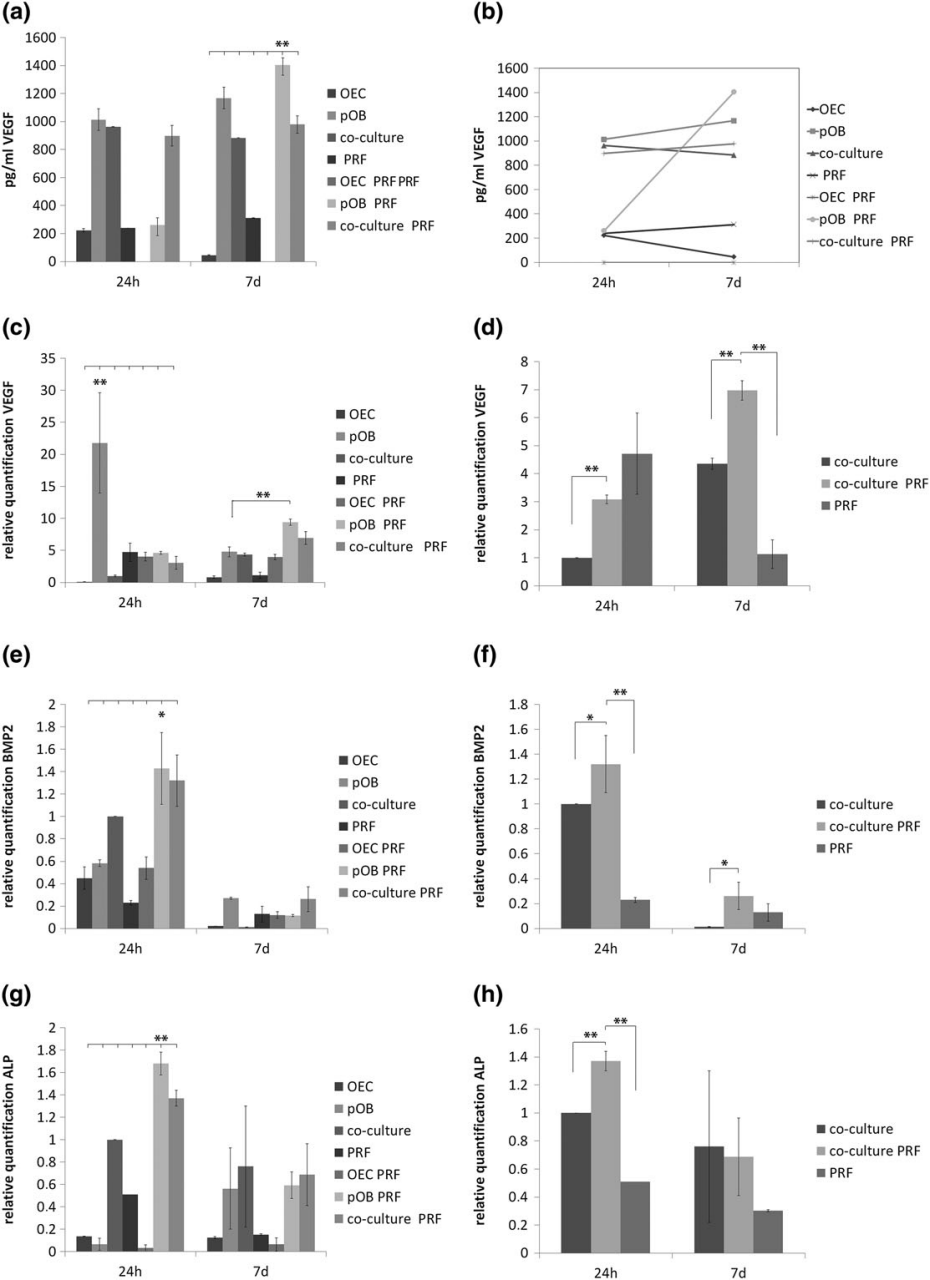
Effect of platelet‐rich fibrin (PRF) on vascular endothelial growth factor (VEGF) expression, VEGF protein content, bone morphogenic protein 2 (BMP‐2) expression and alkaline phosphatase (ALP) expression. VEGF protein amount in cell culture supernatants (a, b) was determined using enzyme‐linked immunosorbent assay (ELISA) and relative gene expression levels of VEGF was analysed by quantitative real‐time polymerase chain reaction (PCR) using the ΔΔCt method and setting (c, d). BMP‐2 expression, as well as ALP expression, was analysed using quantitative real‐time PCR (e‐h). The analysis was performed in iPRF clots compared with PRF mixed either with outgrowth endothelial cells (OECs), primary osteoblasts (pOBs) or co‐cultures of both cell types. The clot/cell mixture was cultivated for 24 h and 7 days. Statistical significance was achieved when **p < 0.03). Scale bar: 75 μm (d). *n* = 3

VEGF is of considerable importance during wound repair, as the process of angiogenesis is markedly disturbed in non‐healing wounds. During this study, the VEGF protein content was evaluated in supernatants of the different experimental groups after 24 h and after 7 days of cultivation using ELISA (Figure 5a, b). However, the highest VEGF concentration was found in supernatants of pOBs that were mixed with PRF and cultivated for 7 days (Figure 5a). This observation was found to be statistically significant compared with all other experimental groups, as indicated in the relevant figure. Although commonly high VEGF protein secretion could also be detected in monocultures of pOB as well as in co‐cultures with and without mixture with PRF (Figure 5a), the supernatants of pOBs in combination with PRF revealed an intense increase in VEGF protein concentration from 24 h to 7 days of cultivation (Figure 5b). In contrast to this clear increase of VEGF protein concentration in supernatants of pOB/PRF clot complex from 24 h to 7 days of cultivation, the VEGF protein content in supernatants of all other experimental groups did not changed markedly from 24 h to 7 days of cultivation, but rather maintained a similar protein level during the whole course of cultivation (Figure 5b). In addition, the relative gene expression of VEGF was also determined in the different experimental groups using quantitative real‐time PCR (Figure 5c, d). After 24 h of cultivation the gene expression level of VEGF was significantly upregulated in pOB monocultures after 24 h and compared with VEGF expression in all other experimental groups, but decreased from 24 h to 7 days of cultivation (Figure 5c). For the 7 day time point, when comparing all groups, an upregulation of VEGF could be observed when pOBs were cultivated with PRF, which was consistent with the results achieved from ELISA analysis (Figure 5c). For a better visualization, gene expression levels of VEGF in co‐cultures compared with VEGF expression in PRF/co‐culture complexes are additionally highlighted in Figure 5d. VEGF gene expression was significantly upregulated in PRF/co‐culture complexes during the course of cultivation from 24 h to 7 days (Figure 5c).

### 3.4 Effect of injectable PRF on osteogenic differentiation capacity in the co‐culture system

Due to the fact that a tissue‐engineered construct for bone tissue‐engineering purposes should optimally combine a fast connection to the vasculature with a good osteogenic differentiation capacity, analysing the effect of PRF on osteogenic differentiation factors in the PRF/cell complex constituted another point of interest during this study. Bone formation is strongly under the control of the interaction of bone cells and endothelial cells in a healing area and synergistic effects of BMP‐2 and VEGF influencing both angiogenesis and osteogenesis are well known. The gene expression level of BMP‐2, one key regulator of early osteogenesis, was analysed in the PRF/cell complexes after 24 h and 7 days (Figure 5e, f). In general, BMP‐2 expression was higher after 24 h of cultivation compared with the 7 day time point, with the highest expression when pOB monocultures were cultivated in PRF (Figure 5e). Comparing the expression of BMP‐2 in co‐cultures alone with BMP‐2 expression in co‐culture/PRF complexes revealed a significantly higher expression of BMP‐2 when co‐cultures were cultivated in PRF for both time points of cultivation (Figure 5f). In addition, expression of ALP activity, an early osteogenic differentiation marker, was also determined at the mRNA level in the different experimental groups (Figure 5g, h). The highest ALP expression could be found in pOB/PRF complexes after 24 h of cultivation compared with all other groups (Figure 5g). Expression of ALP was found to be significantly higher in co‐cultures cultivated in PRF for 24 h compared with ALP expression in co‐cultures alone (Figure 5h).

## 4 DISCUSSION

During this study, the effect of injectable PRF was analysed in the context of regeneration processes on an *in vitro* co‐culture model system for bone tissue consisting of OECs and pOBs. An ongoing challenge for tissue‐engineering applications is the sufficient vascularization of an engineered construct that is essential for the survival of the implant and an adequate wound repair. Therefore, the generation of a tissue‐like vascularized scaffold for implantation using cell‐based prevascularization strategies in different experimental settings has been documented as advantageous (Amini, Xu, Chidambaram, & Nukavarapu, 2016; Grellier et al., 2009; Rouwkema et al., 2006; Tabata et al., 1999). In the context of bone tissue engineering, co‐culture systems consisting of endothelial cells and osteoblasts or their precursors can be used from different sources in scaffold‐free approaches as well as in combination with a material or co‐implanted in Matrigel®‐plugs (Fuchs, Jiang, et al., 2009; Rouwkema et al., 2006; Stahl et al., 2004). Notably, the co‐culture system of OECs and pOBs has already been described in numerous studies and has been established as a highly beneficial tool to study regeneration mechanisms *in vitro* similar to the *in vivo* situation (Fuchs, Dohle, Kolbe, & Kirkpatrick, 2010; Fuchs et al., 2007; Fuchs, Ghanaati, et al., 2009; Fuchs, Jiang, et al., 2009). Furthermore, this *in vitro* model allows the identification of factors that might have a positive effect on processes involved in wound repair, in particular angiogenesis (Dohle et al., 2010; Dohle et al., 2011; Dohle et al., 2014; Li et al., 2014; Ma, Dohle, Li, & Kirkpatrick, 2017). Although the detailed interaction of endothelial cells with osteoblasts in this established co‐culture system underlies complex regulatory mechanisms that are still under investigation, microvessel‐like structure formation can be documented in long‐term co‐cultures after 4 weeks of co‐cultivation (Herzog, Dohle, Bischoff, & Kirkpatrick, 2014). An earlier angiogenic response could only be achieved when co‐cultures were treated with external proangiogenic stimuli like growth factors, morphogens or the addition of other cell types (Dohle et al., 2010; Dohle et al., 2011; Dohle et al., 2014). The aim of this study was to mimic the physiological process of wound repair *in vitro* using PRF concentrates as a natural growth factor‐producing wound healing matrix, and to test if simply embedding the established co‐culture system consisting of pOBs and OECs in PRF might lead to a positive effect of wound healing‐associated processes with regards to the angiogenic activation of the OECs in this system. To the best of our knowledge, this is the first *in vitro* study that combines the preparation of autologous PRF concentrates as a natural scaffold with a co‐culture system for bone tissue consisting of primary cells. Although the detailed mechanisms are not fully understood and systematic *in vitro* as well as *in vivo* studies are necessary to further investigate cell and tissue reaction in response to PRF, the results show that combining the established co‐culture system of OECs and pOBs with injectable PRF primes the angiogenic activation of OECs in the generated matrix and is accompanied by a significant increase in VEGF expression as well as a higher expression of wound healing‐associated factors.

Historically, the development of platelet‐rich plasma products started in the early 1980s, but was first presented to dentists by Whitman and colleagues in 1997, who assumed that the activated platelets and their release of growth factors would improve healing processes after surgery (Chow, McIntire, & Peterson, 1983; Delaini, Poggi, & Donati, 1982; Whitman, Berry, & Green, 1997). Furthermore, the development of leukocyte‐ and platelet‐rich‐fibrin (L‐PRF) was pointed out as the first blood‐derived PRF matrix without using anticoagulants and as a potential target for complex tissue engineering (Choukroun et al., 2001; Choukroun et al., 2006a). Since then, PRF‐producing protocols have evolved, and include the reduction in *g*‐force or changes in the centrifugation time, with the aim of permanently optimizing the PRF clots with regard to cell distribution, growth factor expression and fibrin matrix density (Ghanaati et al., 2014). However, leukocytes as well as the fibrin matrix play a key role during the early stages of the wound repair process (Litvinov & Weisel, 2016). In general, wound healing consists of four overlapping phases, i.e. haemostasis, inflammation, proliferation and new tissue formation (Eming, Brachvogel, Odorisio, & Koch, 2007; Eming, Krieg, & Davidson, 2007; Gosain & DiPietro, 2004). When tissue injury occurs, activated platelets accumulate at sites of injury and form a fibrin clot, which serves as a wound matrix and closes the wound. These activated platelets of the fibrin clot secrete different growth factors, such as PDGF or transforming growth factor ß (TGFß) and are therefore essential for the initiation of the inflammatory process by mediating the recruitment and activation of immune cells (leukocytes) (Martin, 1997). Although PDGF is also expressed in endothelial cells and activated macrophages, this mitogen is mainly synthesized and stored in alpha‐granules of platelets (Heldin, 1992). According to a current study of Choukroun and Ghanaati (2017), who recorded a very high number of activated platelets and leukocytes in injectable PRF produced by LSCC, the PDGF expression was higher in pure PRF compared with control monocultures after 24 h, indicating the existence of a high amount of platelets and leukocytes in the PRF concentrates used for this study. Interestingly, PDGF expression was compared in co‐cultures with and without combination with PRF and was found to be highly upregulated in co‐culture/PRF complexes after 24 h and after 7 days of cultivation. The aggregated platelets from the fibrin clot express a wide array of cell surface immune receptors and contain granules with a number of growth factors and immune mediators (Jenne, Urrutia, & Kubes, 2013) and are therefore able to activate, participate and modulate the host immune response (von Hundelshausen & Weber, 2007). The upregulation of PDGF in co‐cultures combined with PRF might indicate the initiation of wound repair processes in the co‐culture/PRF clots meditated through LSCC prepared PRF. Although it is not generally possible to distinguish exactly which cell type is producing which amount of growth factor in this system, we might assume from this result that a higher expression of PDGF in PRF/co‐culture complexes could be a consequence of the high content of platelets and leukocytes in the PRF clot (Choukroun & Ghanaati, 2017). Furthermore, results from PDGF protein secretion determination evaluated using ELISA revealed a clear trend of producing significantly more PDGF when co‐cultures were mixed with PRF, which additionally confirm the assumption. In addition, we could also assess the relative gene expression profiles of adhesion molecules that are strongly involved in the process of wound healing, in particular in the proceeding inflammation. E‐selectin, as well as ICAM‐1 expression, was significantly upregulated in co‐cultures combined with PRF compared with the expression of these adhesion molecules in the co‐culture without PRF, thus indicating a proinflammatory effect of PRF on the established co‐culture system. From the literature it is known that E‐selectin and ICAM‐1 are strongly involved in the interaction between endothelial cells and leukocytes during the process of inflammation (leukocyte rolling) (Abbassi, Kishimoto, McIntire, Anderson, & Smith, 1993). As expected, E‐selectin, also named endothelial‐leukocyte adhesion molecule, is more highly expressed on OECs, in co‐cultures of pOBs and OECs as well as in their combination with PRF. Determining E‐selectin protein production revealed a high secretion of E‐selectin from PRF clots alone and also when cells were combined with PRF, indicating an ongoing inflammatory stimulus mediated through PRF. Leukocyte‐endothelial cell adhesion is further mediated through ICAM‐1 to a stronger extent before leukocytes transmigrate and initiate the proliferative phase of wound healing (Fernandez‐Borja, van Buul, & Hordijk, 2010; Libby, 2002). Comparing ICAM‐1 expression among the different experimental groups revealed a similar gene expression profile, a higher expression in OECs and co‐cultures as well as in their combination with iPRF, but less expressed in pOB monoculture. Due to the fact that ICAM‐1 is also expressed by leukocytes, it was also found in pure injectable PRF (Thomson et al., 1999).

When considering the immunohistochemical staining for the endothelial cell‐specific marker CD31, the high relevance for PRF for tissue‐engineering applications becomes even more evident by the observation that PRF in combination with the co‐culture of pOBs and OECs leads to an angiogenic activation of the OECs in this complex already after 7 days of complex cultivation *in vitro*. OECs clearly form luminal structures composed of more than one endothelial cell, which seem to be stably embedded in the PRF matrix. The proangiogeneic effect of PRF concentrates has already been analysed from our group in an *in vivo* setting, indicating a high vascularization in nude mice when PRF was implanted, depending on the fibrin scaffold composition and on the porous structure (unpublished data), but has never been documented in an *in vitro* study up to now. During this study, the formation of microvessel‐like structures could not be found in co‐cultures solely consisting of pOBs and OECs without PRF, indicating the proangiogenic effect of PRF on the OECs. Angiogenesis plays an essential role during the process of wound healing and is mediated by activated leukocytes through release of different growth factors (PDGF, TGFß) that attract fibroblasts to wound sites where they produce extracellular matrix within the granulation tissue (Drinkwater, Smith, & Burnand, 2002; Eming, Krieg, et al., 2007; Martin, 1997; Werner & Grose, 2003). Consistently, the combination of pOBs or co‐cultures of pOBs and OECs with PRF leads to a higher matrix formation and a more stable fibrin network compared with PRF alone or in combination with OECs evaluated morphologically using a histological staining with H&E. Furthermore, fibroblasts and leukocytes produce and secrete VEGF within the defective area, which finally leads to new blood vessel formation (Werner & Grose, 2003). Accordingly, results generated from ELISA estimated the highest VEGF protein concentration in supernatants of PRF combined with pOB monoculture after 7 days of complex cultivation compared with the VEGF concentration in supernatants of all other analysed experimental groups. Although the concentration of VEGF in supernatants of pOBs alone was also consistently high during the course of cultivation, the strongest increase in VEGF protein content could be documented for the PRF/pOB group. This suggests that the VEGF secretion by pOBs might be triggered in the presence of PRF, although it is generally documented that pOBs alone express and secrete VEGF to a high extent (Dohle et al., 2010). Interestingly, immune cells of the fibrin clot accumulate at sites of osteopontin‐positive stained cells (pOBs) evaluated by immunohistochemistry, leading to the assumption that the immune cells within the PRF clot might trigger the VEGF production in the pOBs. The fact that the VEGF concentration in co‐culture/PRF complexes is less than in pOB/PRF complexes might indicate that the OECs within the co‐culture/iPRF complex might take up the free VEGF, which leads to their angiogenic activation. Similar results have already been observed in another study from our group in which macrophages were added as proinflammatory stimulus to the co‐culture system consisting of OECs and pOBs (Dohle et al., 2014). Results from real‐time PCR, determining the gene expression level of VEGF in the different experimental groups, confirmed a significant upregulation of VEGF in co‐culture/PRF complexes after 7 days of cultivation. The synergistic effect of VEGF and BMP‐2 has been documented, especially with regard to angiogenesis and osteogenesis (Peng et al., 2005; Zhang et al., 2014). It is well known that VEGF on the one hand initiates vascular network formation, which in turn enhances bone formation via BMP‐2 (Kempen et al., 2009). Significant upregulation of BMP‐2 in the co‐culture/PRF complexes compared with co‐cultures alone might also document an ongoing osteogenic differentiation process in this system, which strongly points to the close relationship between osteogenesis and angiogenesis. Furthermore, BMP‐2 expression controls ALP expression and osteoblastic mineralization, the authors mean that the results of this study in terms of ALP expression are convenient to the literature convenient to the results of gene expression analysis, documenting a significant upregulation of ALP when co‐cultures were mixed with PRF and cultivated for 24 h (Rawadi, Vayssiere, Dunn, Baron, & Roman‐Roman, 2003). Nevertheless, further studies analysing the detailed mechanisms of VEGF and BMP‐2 in bone regeneration would be highly important for directing possible future clinical applications.

## 5 CONCLUSION

The results presented here make autologous PRF‐based matrices generated by LSCC a very beneficial therapeutic tool by simply profiting from the natural conditions in the human body. Using PRF containing blood plasma, platelets and leukocytes for tissue‐engineering purposes results in the initiation of wound healing processes in the established co‐culture system of pOBs and OECs *in vitro* with special attention to the improvement of the process of angiogenesis. Although this study has to be understood as initial basic research and further investigations are necessarily required, this observation might be of high clinical relevance, providing the opportunity to generate a fully autologous prevascularized scaffold for tissue‐engineering purposes.

## CONFLICT OF INTEREST

The authors have declared that there is no conflict of interest.
